# Modelling and dynamic analysis of slippage level for large-scale skid-steered unmanned ground vehicle

**DOI:** 10.1038/s41598-022-20262-z

**Published:** 2022-09-26

**Authors:** Kang Zhou, Shuaishuai Lei, Xiongzi Du

**Affiliations:** School of Mechatronical Engineering, Beijing Institute of Technology, Beijing, China

**Keywords:** Engineering, Mathematics and computing

## Abstract

To analyze the turning characteristics of a large-scale skid-steered unmanned ground vehicle (UGV), a mathematical model based on geometric characteristics and kinematics was developed in this work. The turning characteristics, as well as some important geometric and moving features, were seriously considered in the model. A self-designed UGV was employed to conduct targeted experiments. Initial experiment showed that the width of UGV was much larger than that of actual value, because slippage phenomenon made the errors of actual velocity and trajectory length between two sides of the UGV were lower than those obtained from direct wheel velocities measurements and calculations. Further experiments which obtained moving velocity of the UAV by means of a highly accurate Inertial Navigation System (INS) were conducted. Combining this velocity, the slippage levels of the four wheels can be quantitatively measured. In addition, using standard velocities derived from the accurate moving velocity, a true turning radius can be accurately calculated in real time when the slippage characteristics were seriously considered, hence, the mathematical model can be sufficiently validated. The work can realize modelling and dynamic analysis and estimation of the slippage characteristics of this type of UGV.

## Introduction

Unmanned ground vehicles (UGVs) is supposed to replace humans for various applications in both civilian and military areas, and have been expected to greatly change human life and land combat forms in the near future^1^. Recently, it has been widely applied on various occasions, such as space, defense, agriculture, exploratory, transportation, mining and construction. During these applications, increasing autonomy can not only improve the safety and convenience, but also enlarge the range and scope of the potential applications^2,3^. One of the key components for these vehicles is the steering system, due to it directly relates to the locomotional system design, trajectory and motion planning, and some special tasks arrangement. Ackerman steering, differential steering, and skid-steering are the most widely employed steering mechanisms for majority of wheeled and tracked ground vehicles. Ackerman steering has many advantages, such as good lateral stability, low power consumption, but it also has the disadvantages of low maneuverability and the requirement for an explicit mechanical steering subsystem^4,5^. Differential steering is always used for vehicles with two wheels. It can provide a high maneuverability with a zero turning radius and has a simple steering configuration. However, it does not have strong traction and mobility over rough and loose terrain, it is seldom used for outdoor terrain^4,6,7^. Like two wheels differential steering, skid-steering has various advantages, such as good maneuverability^8^, faster response^9^, simpler and more robust mechanical structure^5^, in addition, the vehicle can also have more room for mission equipment^10^. Due to strong traction, high mobility and other significant merits, skid-steered vehicles are more suitable for all-terrain traversal, particularly for off-road environments^11,12^.

Unlike Ackerman-steered vehicles, skid-steered vehicles do not need a special mechanical steering subsystem^13^, and are driven by independently changing the velocities of the right and left wheels or tracks. When the wheels or tracks on both sides are rotating with the same speed, the vehicle moves straight ahead, while as soon as wheel or track on one side becomes slower or faster, the vehicle can be forced to rotate around an axis, which is outside or inside of the vehicle^14^. During the moving process, all wheels or tracks remain parallel to the longitudinal axis of the vehicle, and the vehicle turning requires slippage of the wheels or tracks^4^. Compared to other types of vehicles, it can perform zero radius cornering and provide larger draw bar pull^15^. Under these circumstances, the wheels and tracks of the skid-steered vehicle roll and slide at the same time, which can increase difficulties in accurately developing corresponding kinematic or dynamic models to describe the locomotion. Moreover, since it is impossible to predict an exact motion based only on its control inputs, skid-steering kinematics is not straightforward^16^.

However, accurately analyzing the skid-steering performance of the vehicle is so important for trajectory planning and control algorithm design in this type of vehicles^17^. In recent decades, many scholars have published some works about model description for this type of steering mode, both for wheel and track driven. Pentzer et al.^18^ established a model based on the instantaneous centers of rotation of a tracked skid-steering robot, and then derived corresponding velocities on both sides. Two similar works can be found in Wu et al.^19^ for wheeled skid-steering mobile robots and Martínez et al.^9^ for tracked mobile robots. These three works used different methods to identify corresponding parameters to accomplish the models establishment. Yi et al.^20^ developed a kinematic model based on the same principle, and then introduced an inertial frame and used an inertial measurement unit (IMU) for skid-steered mobile robot positioning and wheels slippage estimation. Moreover, Zheng et al.^21^ extended a typical four-wheel model and added the traction force and tangential force, and chose motor voltage as the system input and wheel linear speed as the system output, then a nonlinear equation was obtained to analyze the relations between different velocities and turning radii of the skid-steered wheeled vehicle by means of a special system identification algorithm. To design a higher level path tracking controller, Maalouf et al.^22^ and Wu et al.^23^ used heading angle, and linear and angular velocities to establish a two-dimensional moving model, and then conducted a path tracking control. Combining the rotating characteristics of a single wheel and the instantaneous center of rotation, Dogru et al.^24^ proposed a kinematic model for a skid-steered wheeled platform. Wang et al.^25^ simplified a skid-steel wheeled vehicle to a single-axle model, and then derived the velocities, sideslip angles and angle velocities of the wheels on both sides. They believed that it was enough to do local path planning. To analyze characteristics of a skid-steering vehicle, Ren et al.^26^ first provided a kinematic model based on calculating the moving velocities of the four wheels, and then various dynamic characteristics were added to accomplish the corresponding dynamic analysis. Combining a special simulation tool and corresponding experiments, more important dynamic characteristics could be obtained. Although the above works focused on kinematic or dynamic modelling for skid-steered vehicles, both using track and wheel driving, and both about large-scale and small-scale vehicles, the models lacked intuition and cannot be directly related to the establishment of model and experimental deduction. Moreover, the models cannot sufficiently and accurately estimate slippage characteristics, which limited their capacities to service the actual further analysis and design.

In addition, for a tracked mobile robot, Moosavian et al.^27^ established a kinematic model using velocity and rotation information, and then presented a relation between the self-designed slip coefficient and path curvature. Then, actual moving information of the robot was obtained using a laser range finder with the corresponding algorithm. Finally, the model was validated by trajectory planning and control experiments. Additionally, Wang et al.^28^ developed a set of kinematic models for four generic wheel mobile robots(WMRs) to explore the behaviors and characteristics of the robots in the presence of wheel skidding and slipping. The kinematic model was employed to understand the formulation of disturbance perturbations and associated properties. However, the first work focused on a small-scale tracked vehicle, actual moving information was not highly reliable, and the slip coefficient model lacked sufficient theoretical derivation. The second work concerned theoretical deduction from control design perspective and so many parameters were involved in the model, which made the model so complex and might not be convenient to be applied in reality. In addition, the work also lacked reliable experimental validation.

According to a comprehensively review of previously relative published works, it can be concluded that there were so few effective models to describe a large-scale skid-steered four-wheel drive UGVs. For this type of vehicle which cannot be moved by a person and the weight is above 200 kg, the slippage characteristics are so important because the size and the weight are large, small slippage may have significant influences for the moving feature, which may seriously affect the moving and other relative characteristics, such as localization, dead-reckoning, tracking controlling, of the vehicles^29,30^.

In this work, a mathematical model of a large-scale skid-steered four-wheel drive UGV was established to further explore the slippage characteristics, and then quantitatively estimated the slippage level. The model was first established based on the geometric features of the vehicles, and then the influence of the slippage phenomena was induced and further analyzed. Corresponding experiments combining other relative instruments were conducted to assist and validate the theoretical deduction. The slippage levels for the four wheels can be quantitatively measured. It is anticipated that this work can strengthen the understanding of the slippage characteristics of this type of vehicle, and then service kinds of academic research or types of applications in reality.

## Mathematical model of the large-scale skid-steered four-wheel drive UGV

In this section, a mathematical model of the large-scale skid-steered four-wheel drive UGV was established. The model was based on overall geometric characteristics and kinematics of the vehicle. First, to make the modelling process convenient and easy to understand, some hypotheses were considered as follows based on special characteristics of this type of UGV:

1. The center of gravity(CG) was at the center of the geometry, and the UGV was bilaterally symmetrical;
2. The whole UGV was rigid, and the deformations of the suspension and tires were not considered;
3. The UGV was moving on a hard and horizontal ground surface, and the four wheels were always in contact with the surface;
4. The relative positions between the four wheels and the body of the vehicle were fixed all the time.

**Figure 1:**
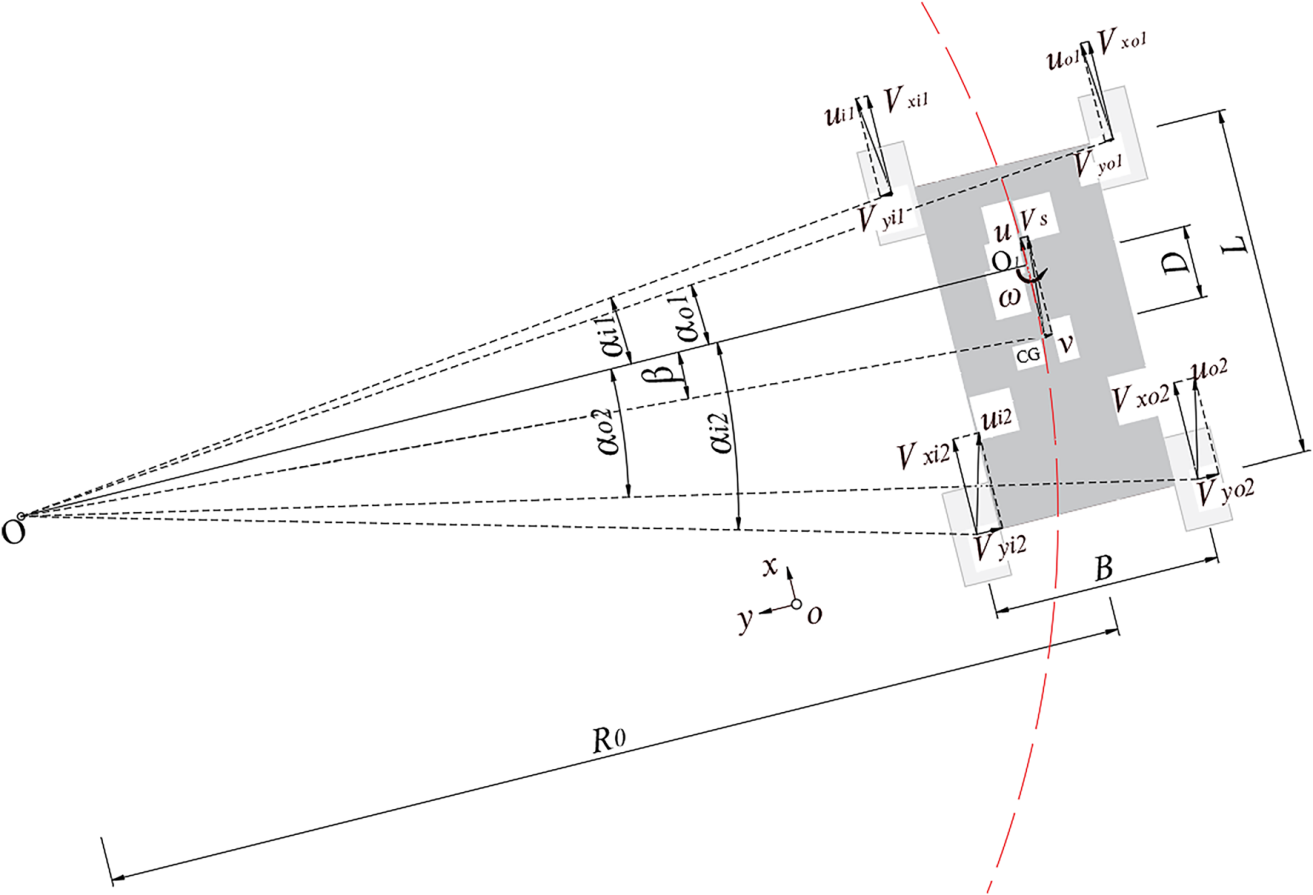
Schematic of the skid-steered four-wheel drive UGV.

Figure 1 shows a schematic of a typical skid-steered four-wheel drive UGV. In this work, the left turning of the vehicle was taken as an example to establish the model. In addition, because this was a comparatively large-scale UGV, we assumed that the turning radius was much larger than the length of the UGV, which could make corresponding analyses more intuitive and convenient.

In Fig. 1, a Cartesian frame (*x, y*) was introduced to describe the initial state of the UGV, and the forward direction of the UGV followed the *ox* axis direction. The length and width of the UGV were respectively denoted as *L* and *B*. If the velocity difference between left and right wheels were unchanged, the whole of the UGV can run follow a circle, on the other hand, the UGV can do self-rotation around a point, so that the forward moving direction was changing all the time. To clearly and conveniently describe this situation, some auxiliary and visual points were introduced, *O* was the center of rotation, *O*_1_ was the projection point of the center of rotation *O* on the center of the UGV, and this point was also the center of self-rotation of the UGV. Obviously, the distance between *O* and *O*_1_ was the radius of the rotation and marked as *R*_0_. Under these circumstances, *O*_1_ was much closer to the front of the UGV than that of *O*^31^, and the distance between *O*_1_ and CG was marked as *D*, so its value range was between 0 and *B*/2. For the locomotive system description, the forward velocity of the UGV along the *ox* axis direction was *u*, while the lateral velocity was *v*. These two velocities can be synthesized to be a new velocity, which was marked as “*V*_*s*_”, and the direction was determined by the amplitudes and directions of *u* and *v*. In addition, there were four wheels presented in Fig. 1, and different wheels were marked using different combinations of subscripts. Subscripts “*i*” and “*o*” respectively denoted inner and outer wheels, and they respectively corresponded to the left and right wheels, while subscripts “1” and “2”, respectively denoted front and rear wheels. There were three velocities associated each wheel. The first was the longitudinal velocities of the wheels, which were *V*_*xi*1_, *V*_*xo*1_, *V*_*xi*2_ and *V*_*xo*2_, respectively described the corresponding velocities of inner-front wheel, outer-front wheel, inner-rear wheel and outer-rear wheel. Because the UGV turned left during the process, a lateral velocity component existed in each wheel, which can be marked as *V*_*yi*1_, *V*_*yo*1_, *V*_*yi*2_ and *V*_*yo*2_ for four wheels, and it should be noted that under the circumstance shown in Fig. 1, the directions of *V*_*yi*1_, *V*_*yo*1_ were from the wheel to left hand, while the directions of *V*_*yi*2_ and *V*_*yo*2_ were from the wheel to the right hand. In addition, their amplitudes were much smaller than those of the longitudinal velocities of corresponding wheels. Moreover, *u*_*i*1_ denoted the velocity by synthesizing *V*_*xi*1_ and *V*_*yi*1_ for the inner-front wheel, and *u*_*o*1_, *u*_*i*2_ and *u*_*o*2_ had the same meanings for the other three wheels. It should be noted that *u*, *V*_*xi*1_, *V*_*xo*1_, *V*_*xi*2_ and *V*_*xo*2_ had the same direction which was parallel to the length of the UGV, while directions of *v*, *V*_*yi*1_, *V*_*yo*1_, *V*_*yi*2_ and *V*_*yo*2_ were parallel to the width of the UGV, in other words, these two arrays of velocities were perpendicular to each other. Furthermore, the directions of *u*_*i*1_, *u*_*o*1_, *u*_*i*2_ and *u*_*o*2_ were the tangent directions of the corresponding wheel, and respectively perpendicular to the connection lines between *O* and the center of gravity of the corresponding wheel.

Furthermore, a rotation angle velocity that described CG rotating around *O*_1_ was also introduced and marked as *ω*. Additionally, there were five angles, which were *α*_*i*1_, *α*_*o*1_, *α*_*i*2_, *α*_*o*2_ and *β*, and their meanings can be easily noticed from Fig. 1. According to the corresponding geometric understanding and reasoning, following mathematical relations can be obtained:

1 $$\left\{ \begin{array}{l} \beta = arc\tan \frac{D}{{R_{0} }} \hfill \\ \alpha_{o1} = arc\tan\frac{L - 2D}{{2R_{0} + B}} \hfill \\ \alpha_{i1} = arc\tan\frac{L - 2D}{{2R_{0} - B}} \hfill \\ \alpha_{o2} = arc\tan\frac{L + 2D}{{2R_{0} + B}} \hfill \\ \alpha_{i2} = arc\tan\frac{L + 2D}{{2R_{0} - B}} \hfill \\ \end{array} \right.$$

Because the body of the UGV was considered rigid enough, *V*_*yi*1_ and *V*_*yo*1_ had the same amplitude and direction, while the same situation was observed for *V*_*yi*2_ and *V*_*yo*2_. In addition, the angular velocities of the four wheels, and the angular velocities of the CG rotated around *O*_1_, were the same. Hence, this angular velocity *ω* can be mathematically described in following form:

2 $$\omega = \frac{v}{D} = \frac{{V_{yo1} }}{{\frac{L}{2} - D}} = \frac{{V_{yi1} }}{{\frac{L}{2} - D}} = \frac{{V_{yo2} }}{{\frac{L}{2} + D}} = \frac{{V_{yi2} }}{{\frac{L}{2} + D}}.$$

Also, the lateral velocity *v* can be described using *u* and *β*:

3 $$v = u \cdot \tan \beta = \frac{uD}{{R_{0} }}.$$

Combining Eqs. (2, 3), the velocities along the *oy* axis direction of the four wheels can be obtained:

4 $$\left\{ \begin{array}{l} V_{yo1} = V_{yi1} = \frac{L - 2D}{{2R_{0} }} \cdot u \hfill \\ V_{yo2} = V_{yi2} = \frac{L + 2D}{{2R_{0} }} \cdot u \hfill \\ \end{array} \right..$$

Then the velocities of the four wheels along the *ox* axis direction can be obtained based on Eq. (4), and corresponding geometric relations are as follows:

5 $$\left\{ \begin{array}{l} V_{xo1} = \frac{{V_{yo1} }}{{\tan \alpha_{o1} }} = \left( {1 + \frac{B}{{2R_{0} }}} \right)u \hfill \\ V_{xi1} = \frac{{V_{yi1} }}{{\tan \alpha_{i1} }} = \left( {1 - \frac{B}{{2R_{0} }}} \right)u \hfill \\ V_{xo2} = \frac{{V_{yo2} }}{{\tan \alpha_{o2} }} = \left( {1 + \frac{B}{{2R_{0} }}} \right)u \hfill \\ V_{xi2} = \frac{{V_{yi2} }}{{\tan \alpha_{i2} }} = \left( {1 - \frac{B}{{2R_{0} }}} \right)u \hfill \\ \end{array} \right..$$

It can be seen that *V*_*xo*1_ = *V*_*xo*2_ and *V*_*xi*1_ = *V*_*xi*2_, to clearly present in following statements, *V*_*xo*_ and *V*_*xi*_ were respectively introduced to denote the velocities of the inner and outer wheels along the *ox* axis direction. Hence, the velocity differences between wheels on both sides and the CG can be described as follows:

6 $$\left\{ \begin{array}{l} \Delta V_{xo} = V_{xo} - u = \frac{uB}{{2R_{0} }} \hfill \\ \Delta V_{xi} = V_{xi} - u = - \frac{uB}{{2R_{0} }} \hfill \\ \end{array} \right..$$

Based on Eq. (6), these two differences of velocities had the same amplitude but different directions, and the UGV should turn to the side where velocity was decreased. In this work, it should turn left under the circumstance shown in Fig. 1. To obtain the relation between the velocity difference and turning radius, the amplitude of the velocity difference was denoted as Δ*V*_*x*_. Then the turning radius *R*_0_ can be described as follows:

7 $$\left\{ \begin{array}{l} \Delta V_{x} = \left| {\Delta V_{xi} } \right| = \left| {\Delta V_{xo} } \right| \hfill \\ R_{0} = \frac{B}{{2\Delta V_{x} /u}} \hfill \\ \end{array} \right..$$

According to above mathematical derivation, the following relations can be obtained:

1. When Δ*V*_*x*_ = 0, it meant that there were no velocity differences between wheels on both sides, the UGV was moving straight, at this time *R*_0_ =  + ∞, which meant that the turning radius was infinite and the center of rotation *O* was also at infinity.
2. When Δ*V*_*x*_ = *u* and *R*_0_ = *B*/2, under the circumstances, the velocity of the wheel on the right side was double, while the velocity of the wheel on the left side was 0, and the center of rotation *O* was at the center of right axis of the UGV.
3. When *u* = 0, Δ*V*_*x*_ ≠ 0, the velocities of the wheels on both sides had the same amplitude but different directions, *R*_0_ = *B*/2, and the center of rotation *O* should coincide with *O*_1_.

In addition, *D*, which was distance between *O*_1_ and CG, can be obtained based on analyzing the geometric relation shown in Fig. 1 combining Eqs. (1) and (7), as well as above three relations:

8 $$D = \left\{ \begin{array}{l} R_{0} \cdot \tan \beta = R_{0} \cdot \frac{v}{u} = \frac{vB}{{2\Delta V_{x} }},\quad u \ne 0 \hfill \\ 0,\quad u = 0 \hfill \\ \end{array} \right..$$

*D* may affect the moving characteristics and relative parameter values of the UGV. Therefore, more mathematical analyses were conducted to obtain concrete and detailed influential levels in this work. According to Eq. (2), the lateral velocities of the four wheels can also be written in following form:

9 $$\left\{ \begin{array}{l} V_{yo1} = V_{yi1} = \omega \left( {\frac{L}{2} - D} \right) \hfill \\ V_{yo2} = V_{yi2} = \omega \left( {\frac{L}{2} + D} \right) \hfill \\ \end{array} \right..$$

Combining the geometric relation between the velocities of the four wheels along the *ox* and *oy* directions, which were *V*_*xi*1_, *V*_*xo*1_, *V*_*xi*2_ and *V*_*xo*2_, as well as *V*_*yi*1_, *V*_*yo*1_, *V*_*yi*2_ and *V*_*yo*2_, and the corresponding angle descriptions in Eq. (1), *V*_*xi*1_, *V*_*xo*1_, *V*_*xi*2_ and *V*_*xo*2_ can also be written in following forms:

10 $$\left\{ \begin{array}{l} V_{xo1} = \frac{{V_{yo1} }}{{\tan \alpha_{o1} }} = \omega \left( {\frac{L}{2} - D} \right) \cdot \frac{{2R_{0} + B}}{L - 2D} = \omega \left( {R_{0} + \frac{B}{2}} \right) \hfill \\ V_{xi1} = \frac{{V_{yi1} }}{{\tan \alpha_{i1} }} = \omega \left( {\frac{L}{2} - D} \right) \cdot \frac{{2R_{0} - B}}{L - 2D} = \omega \left( {R_{0} - \frac{B}{2}} \right) \hfill \\ V_{xo2} = \frac{{V_{yo2} }}{{\tan \alpha_{o2} }} = \omega \left( {\frac{L}{2} + D} \right) \cdot \frac{{2R_{0} + B}}{L + 2D} = \omega \left( {R_{0} + \frac{B}{2}} \right) \hfill \\ V_{xi2} = \frac{{V_{yi2} }}{{\tan \alpha_{i2} }} = \omega \left( {\frac{L}{2} + D} \right) \cdot \frac{{2R_{0} - B}}{L + 2D} = \omega \left( {R_{0} - \frac{B}{2}} \right) \hfill \\ \end{array} \right..$$

Correspondingly, Δ*V*_*x*_ under this circumstance can also be described as follows:

11 $$\Delta V_{x} = \frac{{V_{xo} - V_{xi} }}{2} = \omega \cdot \frac{B}{2}.$$

It can be concluded based on Eq. (11) that *D* cannot affect the velocity control for turning. Additionally, the turning radii of the four wheels can also respectively be obtained as follows:

12 $$\left\{ \begin{array}{l} R_{o1} = \frac{{R_{0} + \frac{B}{2}}}{{\cos \alpha_{o1} }} = \left( {R_{0} + \frac{B}{2}} \right) \cdot \sqrt {1 + \left( {\frac{L - 2D}{{2R_{0} + B}}} \right)^{2} } \hfill \\ R_{i1} = \frac{{R_{0} - \frac{B}{2}}}{{\cos \alpha_{i1} }} = \left( {R_{0} - \frac{B}{2}} \right) \cdot \sqrt {1 + \left( {\frac{L - 2D}{{2R_{0} - B}}} \right)^{2} } \hfill \\ R_{o2} = \frac{{R_{0} + \frac{B}{2}}}{{\cos \alpha_{o2} }} = \left( {R_{0} + \frac{B}{2}} \right) \cdot \sqrt {1 + \left( {\frac{L + 2D}{{2R_{0} + B}}} \right)^{2} } \hfill \\ R_{i2} = \frac{{R_{0} - \frac{B}{2}}}{{\cos \alpha_{i2} }} = \left( {R_{0} - \frac{B}{2}} \right) \cdot \sqrt {1 + \left( {\frac{L + 2D}{{2R_{0} - B}}} \right)^{2} } \hfill \\ \end{array} \right..$$

Hence, the value of *D* can affect these four turning radii, and the detailed influence level can be calculated according to the parameters and variables of the UGV obtained from the actual measurements and corresponding experiments.

## Initial experiment and initial slippage test

### The UGV used in this work

**Figure 2:**
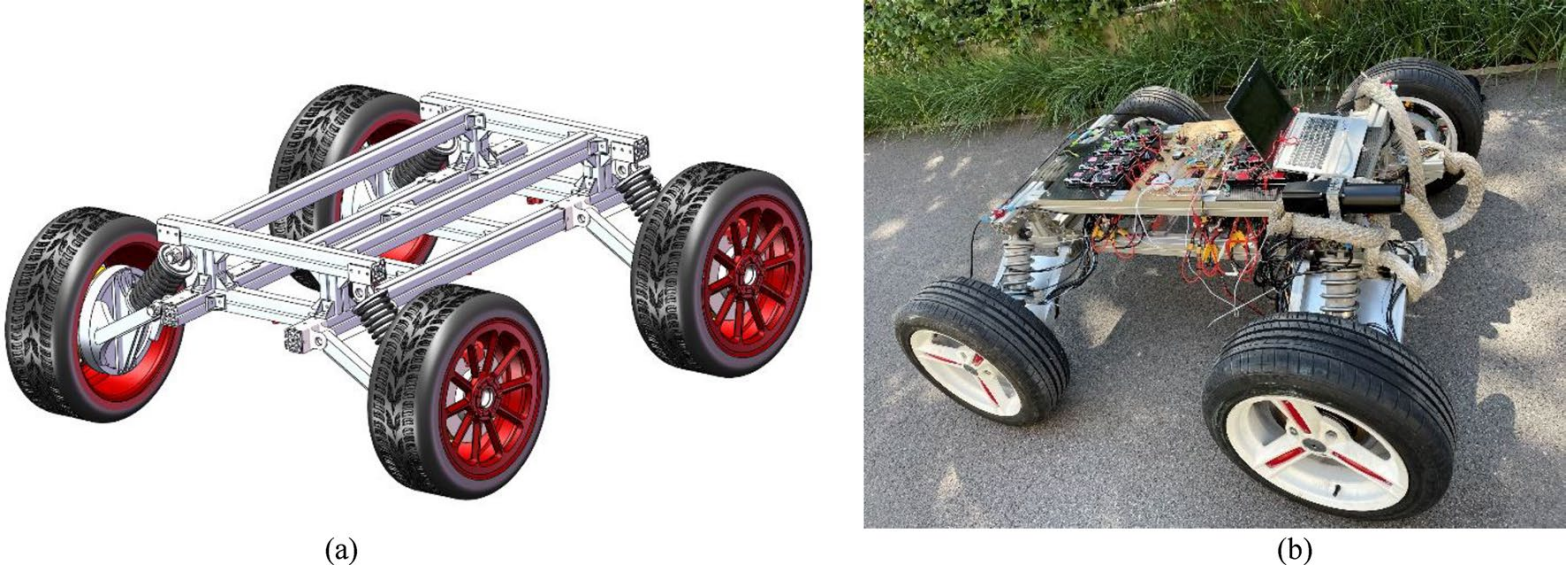
(**a**) A designing diagram using SolidWorks, (**b**) actual UGV used in this work.

For the large-scale skid-steering four-wheel drive UGV, during the turning process, slippage might occur. However, this statement required persuasive experimental data to support. In this work, an actual large-scale skid-steering four-wheel drive UGV was employed to conduct corresponding experiments. This UGV was our self-designed vehicle to execute some special tasks. To make the UGV clearly present, a design diagram using SolidWorks and an actual photo are shown in Fig. 2.

Figure 2a shows the frame of the UGV with four tires, while Fig. 2b shows an integrated UGV with data collection, control, sensing and other auxiliary facilities. This UGV was driven by a series of high-power direct current (DC) motors, and the whole power may exceed 100 kilowatts. Some key parameters of this UGV were shown in Table 1.

**Table 1:** Some key parameter of this UGV.

Item	Whole mass (m/kg)	Length (L/m)	Width (B/m)	Frame height (H_1_/m)	Whole height (H/m)	Radius of each wheel (r/m)
Value	330	1.23	1.45	0.45	0.7	0.31

**Figure 3:**
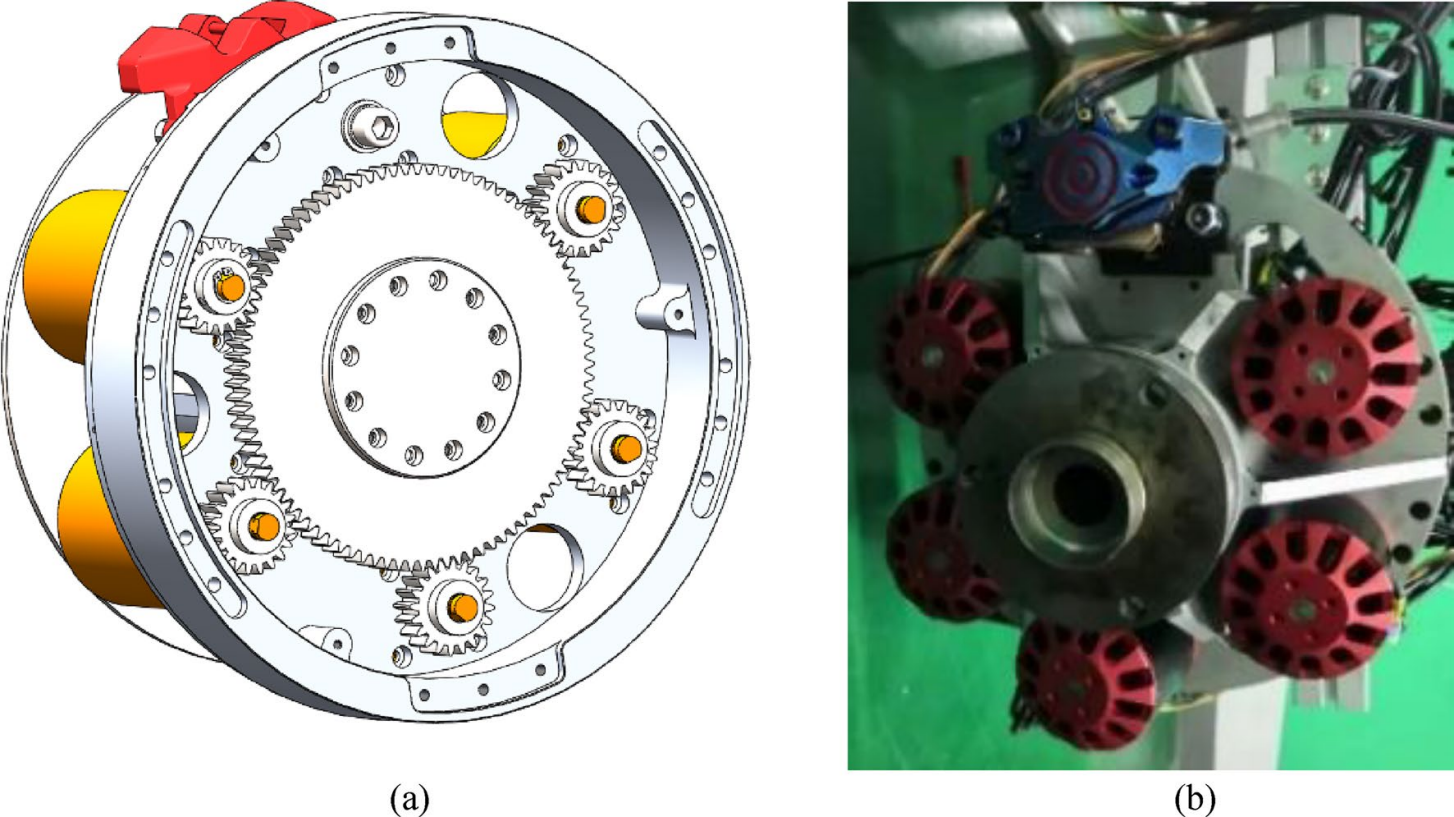
(**a**) Schematic of wheel and motor (outside view), (**b**) actual physical figure of wheel and motor (inside view).

In Table 1, the length and width were measured from the centers of the corresponding wheels. To achieve a high power operation, five DC motors, together with a disc type controllable braking device, were mounted in each wheel hub, corresponding schematic and actual physical figures for one wheel hub were shown in Fig. 3.

For each wheel, five motors were controlled by a Controller Area Network (CAN) bus, so a total of four arrays of this arrangement were included in the UGV. During the moving process, the velocity of one motor in each wheel can be online measured by collecting the frequency of the corresponding inner Hall sensor, and then combining the reduction ratio of the gear and radius of wheel, the moving velocity of this wheel can be accurately obtained.

### Experiment to confirm whether slippage occurring

**Figure 4:**
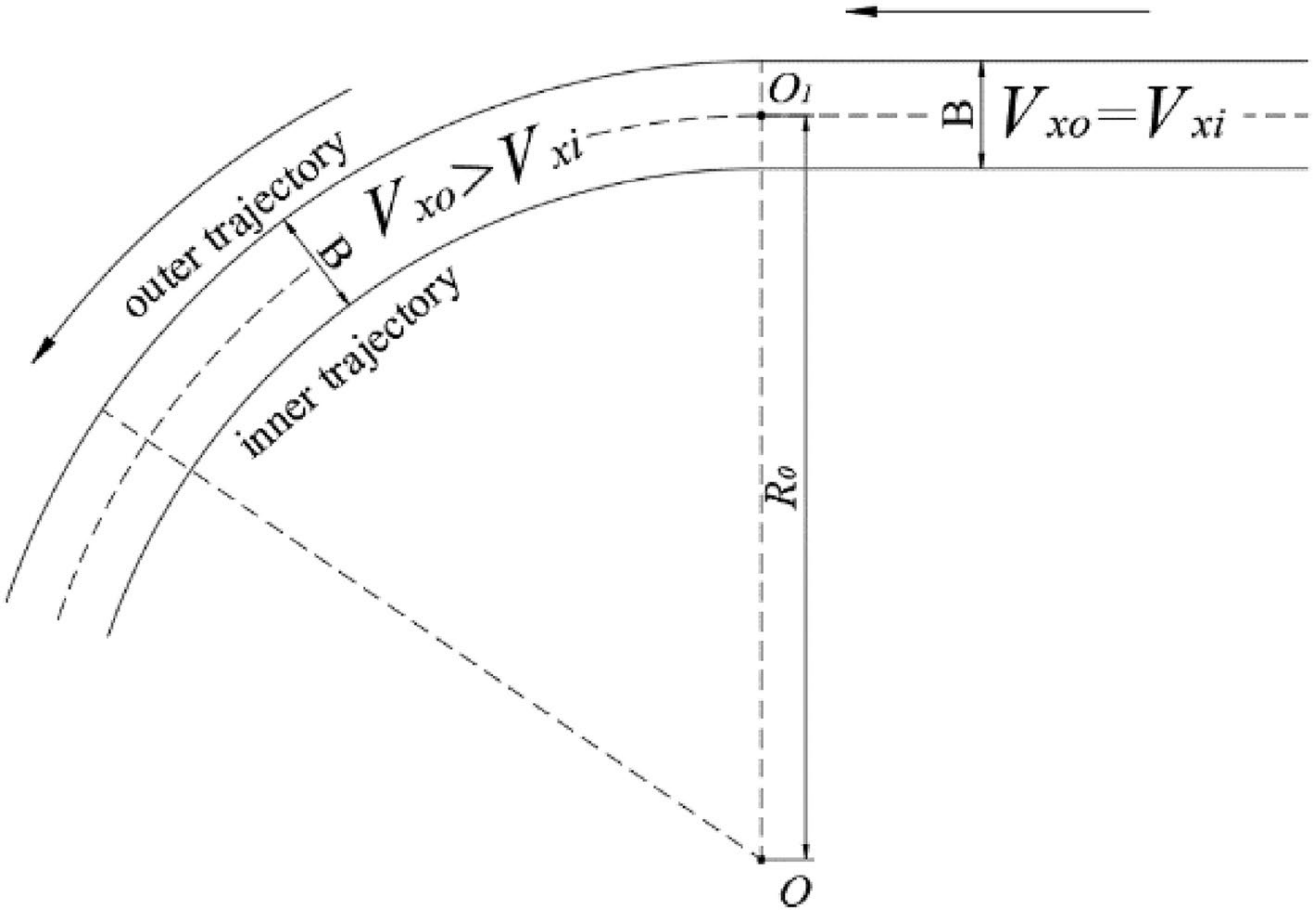
An ideal trajectory of the four wheels during the experiment.

To confirm whether and how much slippage occurred during the turning process, the velocities of four wheels on both sides were set by a special remote controller. The experiment followed the turning direction as shown in “Slippage characteristics analyses and model validation” section, which was turning left. The outer wheels and inner wheels respectively corresponded to right and left wheels. Each experimental process followed two stages: 1. *V*_*xo*_ = *V*_*xi*_, so the UGV moving straight; 2. *V*_*xo*_ > *V*_*xi*_, so the UGV turning left. Therefore, an ideal trajectory of the left and right wheels should be shown in Fig. 4.

As soon as *V*_*xo*_ and *V*_*xi*_, as well as other operational conditions, were unchanged during the second stage, the UGV could run following a standard circle. Moreover, because the width of the UGV, *B*, was a constant during the process, it could be used to validate the model or calculate other variables.

**Figure 5:**
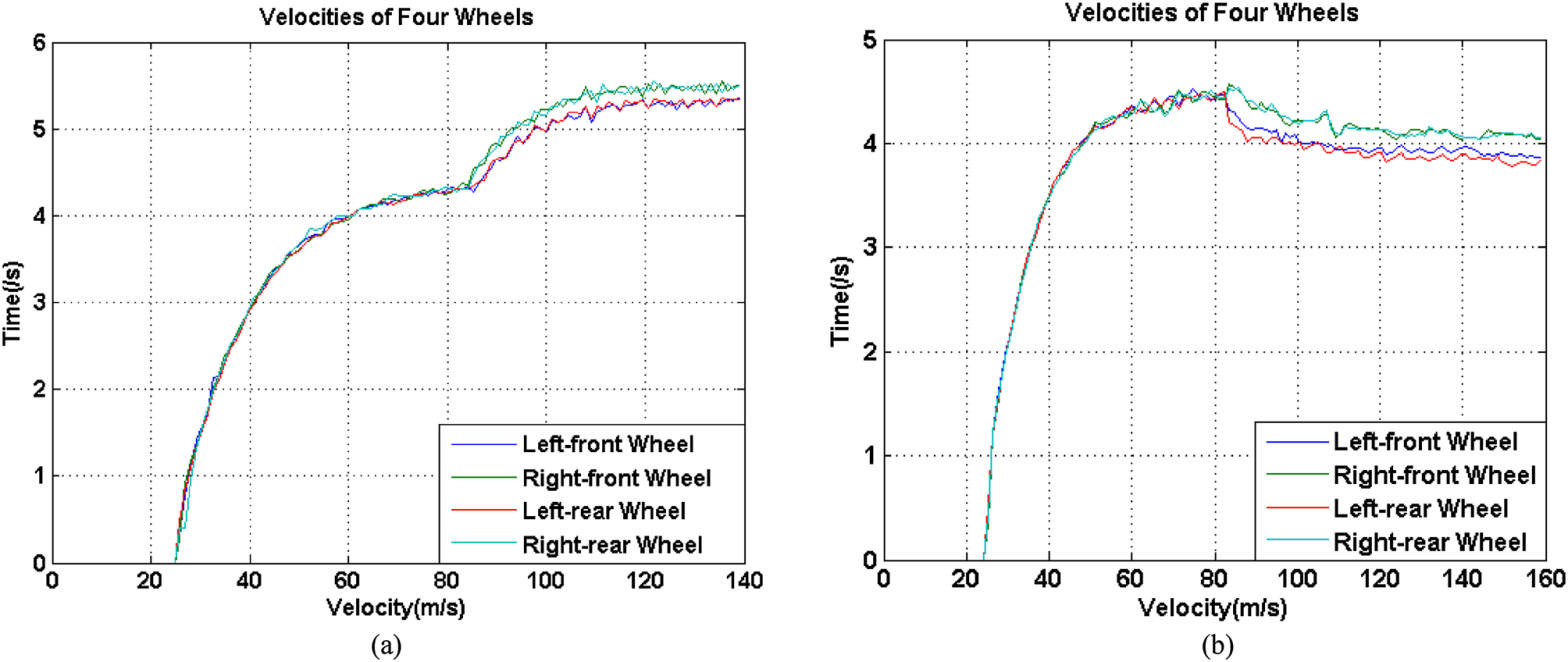
Two arrays of velocities of four wheels: (**a**) Overall velocity increased during the turning process, (**b**) overall velocity decreased during the turning process.

Actual experiments were conducted on a concrete surface. First, the velocities of the four wheels were assured to be simultaneously collected. To make the experimental data more persuasive and clearly presented, two arrays of experimental data, in which one array was obtained when overall velocity increased during the turning process, while the other array was obtained when the overall velocity decreased during the turning process, were chosen. The velocity of these two types of experiments are shown in Fig. 5.

**Figure 6:**
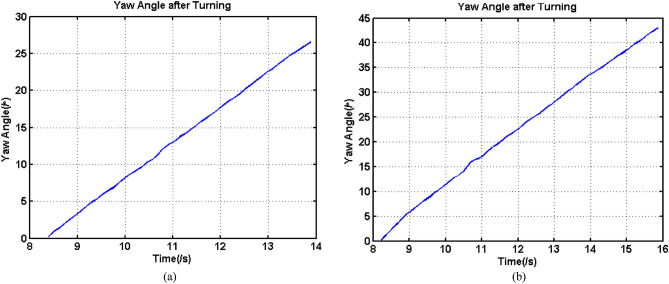
Yaw angle during the turning process, (**a**) Overall velocity increased, (**b**) overall velocity decreased.

The data in Fig. 5a and b shows that the velocities of the four wheels in these two types of experiments had a high degree of consistency, and the velocity variations were so steady, so the data were much better for model calculation and validation. Additionally, an inertial measurement unit (IMU) was mounted at the CG to monitor the variation of central angle, which was a yaw angle of the UGV with a high accuracy, changing with the variation of velocities of wheels during the process. Figure 6 shows the variations of yaw angles of above two experiments during the turning process.

Both of the two yaw angles were increased with two corresponding approximately constant slope, which meant that the differences between *V*_*xi*_ and *V*_*xo*_ of the two experiments were so stable. In addition, it can be observed that during the same duration, the yaw angle variation in Fig. 6b was obviously higher than that in Fig. 6a. It could be derived that the velocities difference between inner and outer wheels in the condition that overall velocity decreased during the turning process was higher than that of overall velocity increased during the turning process, which was coincide with the presentation in Fig. 5a and b.

According to Fig. 3, every stable outer trajectory and inner trajectory can correspond to their individual turning radii, whose error was the width of the UGV, i.e. *B*. Hence, the velocities can be used to calculate the lengths of the outer and inner trajectories, and then combining the variation in the yaw angle, then an actual *B* can be calculated. The corresponding mathematical description is shown in Eq. (13):

13 $$B{ = }\frac{{L_{xo} - L_{xi} }}{{Y\left| {_{s}^{e} } \right.}} = \frac{{\sum_{s}^{e} {V_{xo} t} - \sum_{s}^{e} {V_{xi} t} }}{{Y\left| {_{s}^{e} } \right.}}.$$

The subscripts “*s*” and “*e*” respectively denote starting and ending times of the calculation, *L*_*xo*_ and *L*_*xi*_ respectively are the lengths of the corresponding trajectories, and *Y* denotes the yaw angle variation. The chosen period can be a whole turning process, or a short period with a stable velocity changing. In addition, *V*_*xo*_ and *V*_*xi*_ included two velocities of wheels. To make the calculation more convincing, different velocities were employed to calculate the maximum and minimum errors, as shown in Eq. (14):

14 $$B \in [\min B,\max B] = \left[ {\frac{{\min (L_{xo1} ,L_{xo2} ) - \max (L_{xi1} ,L_{xi2} )}}{{Y\left| {_{s}^{e} } \right.}},\frac{{\max (L_{xo1} ,L_{xo2} ) - \min (L_{xi1} ,L_{xi2} )}}{{Y\left| {_{s}^{e} } \right.}}} \right].$$

Because the above two experimental datasets had stable velocity and yaw angle variations during their turning process, two whole turning processes can be used to calculate *B*. The corresponding calculation results are shown in Table 2.

**Table 2:** Maximum and minimum *B* in moving processes shown in Fig. 5.

Process shown in	min *B* (m)	max *B* (m)
Figure 5a	2.0376	2.2129
Figure 5b	1.9270	2.2259

It can be observed having two features. The first was regardless of the maximum or minimum *B* in the two above processes, it was significantly much larger than the actual width of the UGV, which was only 1.45 m. The second was that comparing the results obtained from two processes, it can be found that the maximum and minimum *B* had very few differences. These two features showed that the calculation processes had no error, but the values of *B* were obviously not reasonable. Combining other similar experimental results, all of the calculated *B* values were remarkably larger than 1.45 m, which meant that the measured difference between the velocities of *V*_*xo*_ and *V*_*xi*_ should be larger than those of the actual values. According to Fig. 4, the actual turning radius *R*_0_ should be the average of radii of the outer trajectory and inner trajectory. However, its value should not be calculated under this circumstance.

According to the above deviation and corresponding calculation, directly using the measured velocities of the wheels cannot obtain the correct and reasonable turning radius and other important information. This meant that the measured velocity, which was collected from the wheels turning, might not be equal to the actual moving velocity of the UGV. The measured differences in velocity and trajectory between the outer and inner wheels were larger than those of actual values, which meant that a part of the turning of wheels might not contribute to the movement of the UGV along the trajectory, in other words, slippage phenomena occurred during the process, which prevented us from obtaining accurate moving velocity and trajectory of the UGV based on online collection of the velocities of wheel turning. To explore the slippage characteristics and quantitative measure and analyze the phenomenon, further and more comprehensive experiments together with advanced calculation methods should be introduced in this work.

## Slippage characteristics analyses and model validation

### Slippage characteristics analyses using a further experiment

During the turning process, the moving velocity of the UGV, i.e. *u* in “Mathematical model of the large-scale skid-steered four-wheel drive UGV” section, should be an average of the true *V*_*xo*_ and *V*_*xi*_. Although the velocities of the four wheels can be measured online using each corresponding built-in Hall sensors, according to previous calculations, a part of wheel turning did not contribute to the forward moving of the UGV, and so the velocity of the wheel should not be equal to the forward moving velocity of the UGV. Hence, to further analyze the slippage characteristics, an accurate forward moving velocity of the UGV should be individually obtained online.

**Figure 7:**
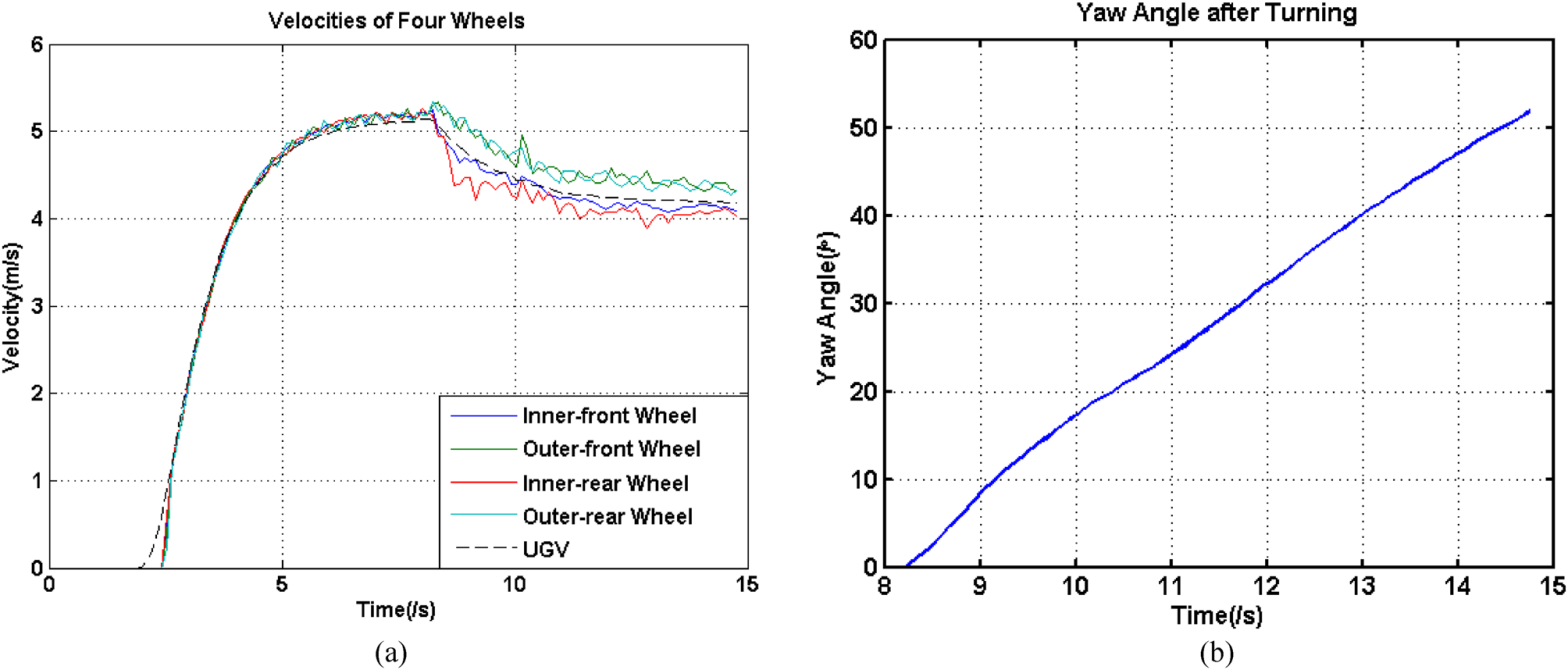
(**a**) Velocities of wheels and UGV, (**b**) yaw angle during the turning process.

According to Fig. 4, the difference between *V*_*xi*_ and *V*_*xo*_ was so small, so the measurement of *u* should have high accuracy. In this work, a highly accurate Inertial Navigation System (INS) was introduced to replace the IMU used in “Initial experiment and initial slippage test” section. The INS can not only supple accurate yaw angle, roll angle, pitch angle, as well as velocities in the north, east and sky directions, but also supply the latitude and longitude information with a high accuracy. Although it can also supply the angle velocity and linear acceleration information, they were not original values and cannot fulfill our high accuracy requirement during a very short period and were abandoned. In this work, reliable velocities in three directions and three attitude angles were employed to calculate the online moving velocity of the UGV and yaw angle. Because the direction cosine matrix(DCM) was obtained directly by three attitude angles instead of angle velocities measured by gyroscopes, and then the forward moving velocity can be calculated by the DCM and collected velocities in three directions, there were no cumulative errors and the results can achieve a high accuracy^32^. Moreover, to guarantee obtaining reliable and highly accurate *u* of the UGV, a series of tests were conducted. Corresponding experiments were conducted and Fig. 7 shows the velocities of four wheels obtained from Hall sensors and *u* obtained from INS, as well as the yaw angle during the turning process.

**Figure 8:**
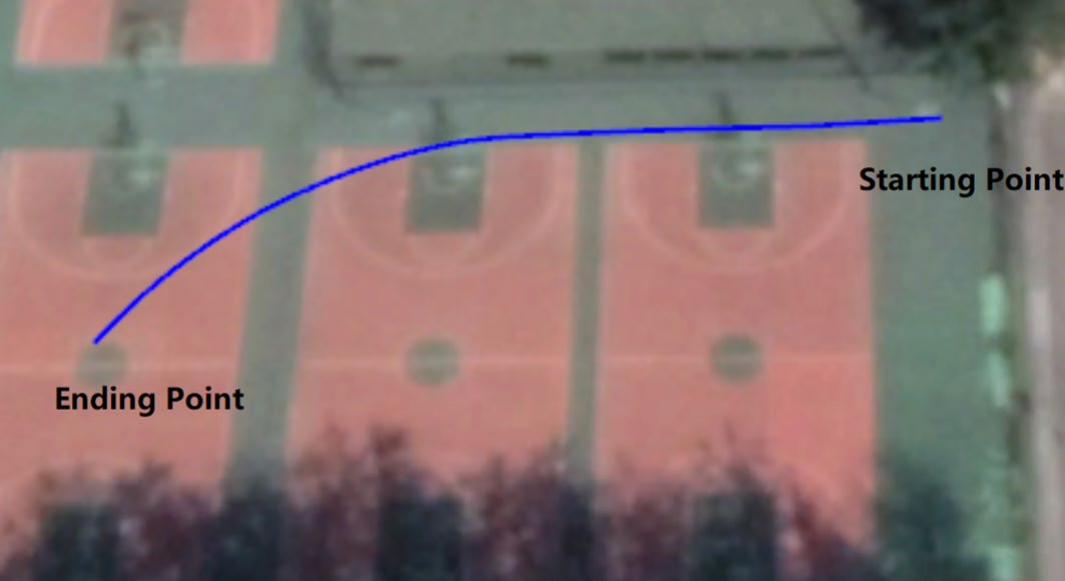
Trajectory description using latitude and longitude variation values.

According to Fig. 7a, the curve of velocity of UGV was in between the curves denoting two velocities of outer wheels and two velocities of inner wheels: *V*_*xo*1_ and *V*_*xo*2_ were larger than *u*, while *V*_*xi*1_ and *V*_*xi*2_ were smaller than *u*. This situation followed our expectation. In addition, to validate the effectiveness of *u*, latitude and longitude information were employed. By means of this information, the curve of the moving trajectory of CG can be depicted as shown in Fig. 8.

It was obviously that the UGV was turning left during the process. In addition, using latitude and longitude information can calculate the length of trajectory, and using *u* can also obtain this information, so the error can be used to validate the effectiveness of the measurement and calculation. In the experiment shown in this part, the two lengths were respectively 53.5145 m and 53.5732 m, and corresponding error was only 0.1578 m with 0.3%. Hence, it can be seen that the measurement of *u* was reliable and accurate enough. Additionally, the yaw angle was obtained using the same instrument. Therefore, it can be said that the forward moving velocity, which was also the longitudinal velocity of the UAV, and yaw angle were reliable and had a sufficiently high accuracy. Based on these two values, the true turning radius *R*_0_ can be correspondingly calculated using Eq. (15):

15 $$R_{0} = \frac{{\sum_{s}^{e} {ut} }}{{Y\left| {_{s}^{e} } \right.}}$$

However, the accuracy of *R*_0_ was determined by the number of collecting points, as well as the moving steadily of the UGV. In other words, each sample point during the turning stage can correspond to one turning radius. To obtain reliable and accurate values, after carefully checking the data, the first and last 5 values of turning radii were abandoned, and the final *R*_0_ was an average of the remaining values.

Under these circumstances, these two parameters can be used to analyze the slippage characteristics of the UGV. Given an accurate value of *u*, combining the model described in “Mathematical model of the large-scale skid-steered four-wheel drive UGV” section and intrinsic parameters of the UGV, accurate trajectories of the outer and inner wheels should be obtained. According to Fig. 7a, it can be seen that during the straightforward moving stage, the velocity of CG, together with the velocities of the four wheels, almost completely overlapped, except in the starting and ending stages. This was because during the starting stage, the Hall sensors had certain errors when detecting a very low movement, while during the ending stage, because the effects of mechanical, assembly and other relative errors on the UGV were much larger, the velocity obtained from INS also had a certain difference when compared to the velocities obtained from the Hall sensor. In addition, according to Fig. 5a, b and Fig. 7a, the velocities of wheels during the turning stage had more vibrations because the wheels might squeeze and twist. Hence, it was unrealistic to use the instantaneous velocities to explore and analyze the moving performance of the UGV in this work.

To avoid inducing errors from mechanical, assembly and other relative errors, the length of trajectory and corresponding mean velocity were employed in this work. Combining true turning radius *R*_0_ obtained from Eq. (15) and the width of vehicle *B*, the standard lengths of the outer and inner trajectories can be obtained:

16 $$\left\{ \begin{array}{l} L_{xo}^{t} = (R_{0} + B/2)Y\left| {_{s}^{e} } \right. \hfill \\ \overline{V}_{xo}^{t} = \frac{{L_{xo}^{t} }}{t} \hfill \\ L_{xi}^{t} = (R_{0} - B/2)Y\left| {_{s}^{e} } \right. \hfill \\ \overline{V}_{xi}^{t} = \frac{{L_{xi}^{t} }}{t} \hfill \\ \end{array} \right..$$

where $L_{xo}^{t}$ and $L_{xi}^{t}$ respectively denote the standard lengths of outer and inner trajectories obtained from theoretical deviation and corresponding calculation based on *u* obtained from INS. $\overline{V}_{xo}^{t}$ and $\overline{V}_{xi}^{t}$, respectively denoted the corresponding mean velocities of outer and inner wheels during the turning process. In addition, for four wheels, there were corresponding trajectories and mean velocities, so they can use the same donation in this work. Based on the initial experiment shown in “Experiment to confirm whether slippage occurring” section, the difference between the outer and inner trajectories calculated by Hall sensors in DC motors of wheels, was larger than those by derived by *u* and *B*. Additionally, to validate the accuracy of the calculation, the width of the UGV was again calculated using the results of Eq. (16), which can validate whether the trajectory after turning left was a standard circle. The calculated width of the UGV in this work was marked as *B*_*c*_. For the actual experiment shown in this part, the corresponding calculation results are shown in Table 3.

**Table 3:** Calculation results of the experiment.

**Data from theoretical deviation and corresponding calculation based on INS**								
Item	*R*_0_ (m)	*B*_*c*_ (m)	$L_{xo}^{t}$ (m)	$L_{xi}^{t}$ (m)	$\overline{V}_{xo}^{t}$ (m/s)	$\overline{V}_{xi}^{t}$ (m/s)		
Value	30.7228	1.4601	29.7085	28.3884	4.4877	4.2883		

**Data from theoretical deviation and corresponding calculation based on actual velocity measurement**								
Item	*L*_*xo*1_ (m)	*L*_*xo*2_ (m)	*L*_*xi*1_ (m)	*L*_*xi*2_ (m)	$\overline{V}_{xo1}$ (m/s)	$\overline{V}_{xo2}$ (m/s)	$\overline{V}_{xi1}$ (m/s)	$\overline{V}_{xi1}$ (m/s)
Value	30.4798	30.5081	27.9008	28.6435	4.6042	4.6084	4.2146	4.3268

**Figure 9:**
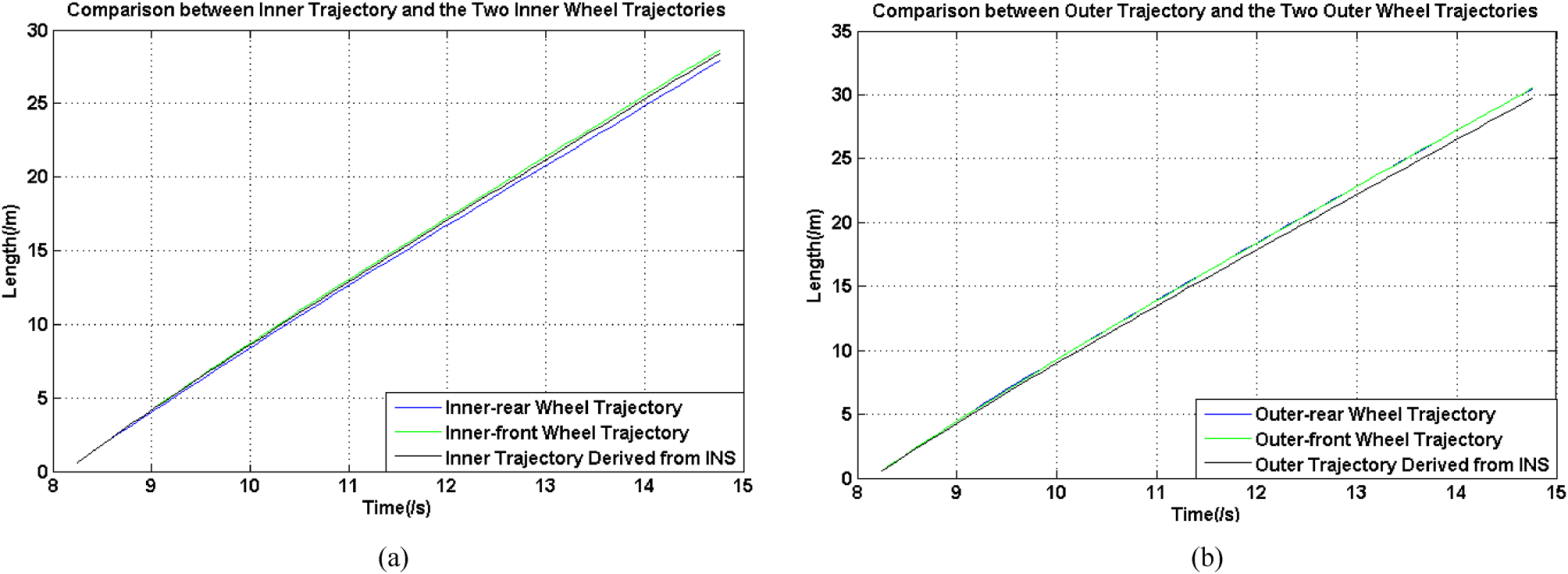
Comparison between trajectories and wheels, (**a**) inner wheels, (**b**) outer wheels.

According to Table 3, the value of the calculated width of the UGV was 1.4601 m, approached the standard width 1.45 m with an error of 0.7%, so the calculation should be reliable enough. Additionally, because the mean velocities were the ratio between the lengths of trajectories and times, the times used in this calculation were the same, the ratios between each mean length and mean velocity are the same in Table 3. In addition, to make the presentation clearer, Fig. 9 shows the comparison between inner and outer trajectories derived from forward moving velocity measured from INS and the two left and right wheels, which were also the inner-rear wheel, inner-front wheel, outer-rear wheel and outer-front wheel under the circumstance.

In Fig. 9a and b, the inner and outer trajectories derived from INS were above other two trajectories, so it was so clear to see the variation of their differences with the moving time. According to Table 3 and Fig. 9, the coincidence between the two trajectories of the two inner wheels was lower than those of the two outer wheels, at the same time, the difference between two trajectories of the two inner wheels and the inner trajectory derived from INS was smaller than those of the two outer wheels. These results can also refer to Fig. 7a, the velocity of the inner-rear wheel had an obvious sudden decrease when the turning process started, and at this time, there was an obvious velocity difference in these two wheels, and from this time on, the differences between trajectory lengths gradually increased. Therefore, for these four wheels, their slippage level should be seriously and individually considered. Under these circumstances, this information can be employed to quantitatively evaluate the slippage levels of the four wheels. For each wheel, the slippage level can be calculated using Eq. (17):

17 $$\left\{ \begin{array}{l} sl_{o1} = \frac{{L_{xo1} }}{{L_{xo}^{t} }},\quad sl_{o2} = \frac{{L_{xo2} }}{{L_{xo}^{t} }} \hfill \\ sl_{i1} = \frac{{L_{xi}^{t} }}{{L_{xi1} }},\quad sl_{i2} = \frac{{L_{xi}^{t} }}{{L_{xi2} }} \hfill \\ \end{array} \right..$$

where *sl*_*o*1_, *sl*_*o*2_, *sl*_*i*1_ and *sl*_*i*2_ are the slippage ratios of two outer wheels and two inner wheels. To make the comparison intuitive, the arrangement of the numerator and denominator were opposite for two outer wheels and two inner wheels, because the standard trajectory length should be smaller than that of the outer wheels, but larger than that of the inner wheels, under the circumstance of slippage occurring. For this experiment, the corresponding four slippage ratios were 1.0260, 1.0269, 1.0175 and 0.9911, respectively for the outer-rear wheel, outer-front wheel, inner-rear wheel and inner-front wheel. It can be quantitatively concluded that the outer wheels had much higher coincidence than the inner wheels. For two inner wheels, one value of slippage ratio was above 1 and the other value was below 1. This may because that all the four wheels were connected to the body of the UGV, for two inner wheels, their theoretical trajectory were significantly affected by the slippage phenomenon. The length of the trajectory of the outer wheels should be larger than that calculated by *u* and *B*, while the item of the inner wheels was smaller than that calculated using the same values. However, not only did the trajectories of the two inner wheels have lower coincidence, but the trajectory of the inner-front wheel was also larger than the $L_{xi}^{t}$. At the end of the straight moving stage, *u* was gradually lower than that measured from the four wheels, which had a high coincidence in this stage. This may be because for this type of large-scale UGV, the mechanical, assembly and other related errors can significantly affect the coincidence of velocity measurement even in the moving straight stage. Second, during the turning stage, two inner wheels encountered more slippage, which can also be found from the velocity curves shown in Fig. 7a. This meant that more movement of the inner-front wheel did not follow the expected trajectory, so its actual trajectory was shorter than the expected length, which meant that this wheel may slide instead of the scrolled during the process. In addition, the two outer wheels also underwent longer trajectories than that of expectance, which meant that some movements were useless and may move toward to other directions.

**Figure 10:**
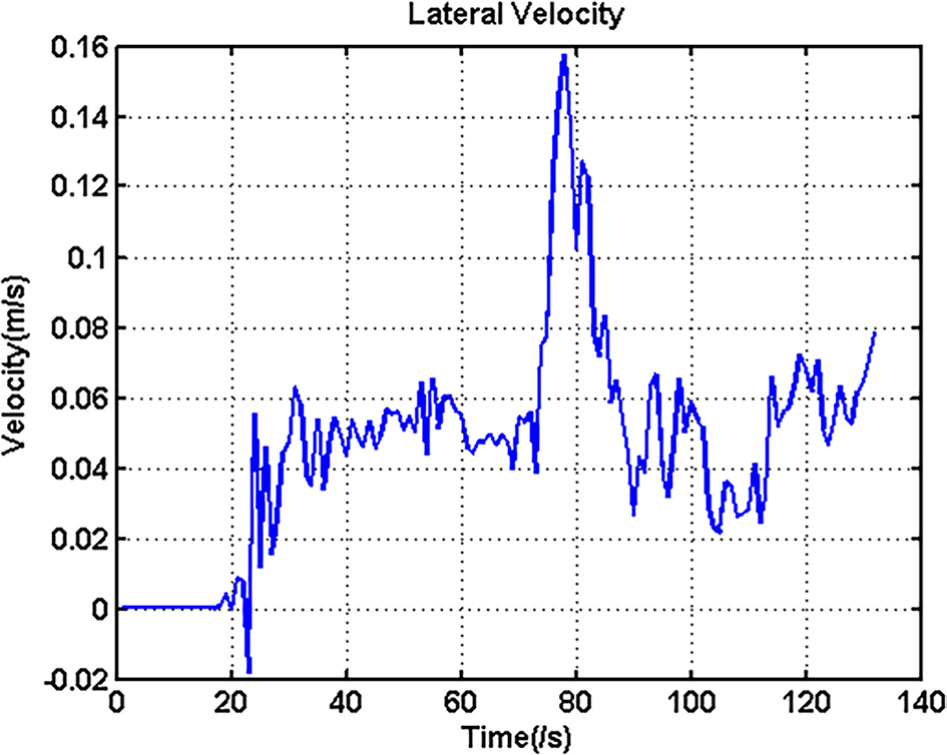
Lateral velocity of the UGV.

In addition, to further explore the turning characteristics of the UGV, lateral velocity *v* should also be considered. Figure 10 shows the lateral velocity variation of this experiment.

According to Fig. 10, it can be seen that during the majority of the moving time, the lateral velocity was very small, and the difference between the velocities during the turning stage and during the moving straight stage was very small. However, at the starting time of the turning stage, the lateral velocity had a significant change, and the maximum value was 0.1572 m/s. When examining Fig. 7a, it can be found that during this time, the inner-rear wheel had an obvious longitudinal velocity vibration. Hence, the maximum lateral velocity should be related to the longitudinal velocity vibration of this wheel.

Moreover, the values of *u*, *v*, and *R*_0_ are known. According to Eq. (8), *D*, which is the distance between the rotation center and geometrical center of the UCG, can be correspondingly calculated. This value was relative to *R*_0_, so the value during the stable state can be calculated using the same method as that of *R*_0_. Then according to Eq. (12), the turning radii of the four wheels can be accordingly calculated. Table 4 shows the values of *D*, and corresponding turning radius of four wheels.

**Table 4:** *D* and Turning Radius of Four Wheels.

Item	*D* (m)	*R*_*o*1_ (m)	*R*_*i*1_ (m)	*R*_*o*2_ (m)	*R*_*i*2_ (m)
Value	0.3999	31.4485	29.9986	31.4642	30.0149

Regardless of whether two outer or two inner wheels were used, the differences in turning radii between the front and rear wheels were very small and could be neglected. Under these circumstances, the trajectories between the front and rear wheels can be considered completely coincident in reality. Actually, even though *D* achieved the maximum value, which was half of *L*, the maximum difference between the turning radii of the front and rear wheels was only 0.025 m, which can also be neglected in reality. Only if *R*_0_ was very small and *D* achieved its maximum should its effect on the four turning radii be considered. Hence, for this type of large-scale UGV employed in this work, it can be considered that this effect can be ignored and that each front and rear wheel on one side can follow the same trajectory.

### Further characteristics analysis using experiments

To obtain more sufficient and convincing conclusions and explore further characteristics of this type of UGV, more experiments were conducted. During the process, the velocities of inner and outer were set to be different so that different turning radiuses were collected, and total 26 arrays of experiments were conducted. Following the same method, some important parameters, such as turning radius *R*_0_, four slippage ratios, which were *sl*_*o*1_, *sl*_*o*2_, *sl*_*i*1_ and *sl*_*i*2_, and calculated width of UGV *B*_*c*_ can be obtained. In addition, the error between lengths calculated by *u* and latitude and longitude information were also recorded and denoted using *E*. To make this error clearer, it was presented in percent form. Additionally, to further reflect the characteristics of velocity differences between the inner and outer wheels, the velocity difference between $\overline{V}_{xo}^{t}$ and $\overline{V}_{xi}^{t}$, as well as the actual velocity difference of the outer and inner wheels, were also calculated and recorded, and their corresponding mathematical descriptions are shown in Eq. (18):

18 $$\left\{ \begin{array}{l} E_{v}^{t} = \overline{V}_{xo}^{t} - \overline{V}_{xi}^{t} \hfill \\ E_{v}^{a} = \frac{1}{2}(\overline{V}_{xo1} + \overline{V}_{xo2} ) - \frac{1}{2}(\overline{V}_{xi1} + \overline{V}_{xi2} ) \hfill \\ \end{array} \right.$$

where $E_{v}^{t}$ and $E_{v}^{a}$ respectively denote above two velocity differences. Table 5 shows the corresponding results, and the experiment mentioned above was included.

**Table 5:** Data record about the UGV turning experiments.

No	*R*_0_ (m)	*sl*_*o*1_	*sl*_*o*2_	*sl*_*i*1_	*sl*_*i*2_	*B*_*c*_ (m)	*E* (%)	$E_{v}^{t}$ (m/s)	$E_{v}^{a}$ (m/s)
1	11.2985	1.1047	1.0669	1.2236	0.9913	1.1538	0.9117	0.5341	1.2874
2	12.9834	1.0572	1.0581	1.0963	0.9655	1.5341	− 0.2020	0.4184	0.7611
3	14.0194	1.0538	1.0527	1.0858	0.9723	1.5311	1.3891	0.4072	0.7380
4	25.3851	1.0276	1.0262	1.0178	0.9828	1.6347	− 0.2399	0.2418	0.3630
5	26.1547	1.0299	1.0294	1.0141	0.9807	1.6258	0.6321	0.2324	0.3523
6	26.7206	1.0194	1.0230	1.0271	0.9948	1.5958	0.6325	0.2109	0.3397
7	27.2132	1.0267	1.0294	1.0260	0.9937	1.4611	− 0.3624	0.2108	0.3684
8	27.2542	1.0265	1.0291	1.0152	0.9858	1.5661	− 0.3080	0.2176	0.3396
9	27.7177	1.0232	1.0304	1.0186	0.9902	1.4796	0.9608	0.1984	0.3229
10	27.9809	1.0313	1.0410	1.0098	0.9813	1.4523	− 0.6806	0.1953	0.3222
11	29.8723	1.0388	1.0389	1.0037	0.9765	1.4841	2.8261	0.2037	0.3365
12	30.6805	1.0240	1.0253	1.0205	0.9869	1.5378	− 0.4181	0.1885	0.3061
13	30.7228	1.0260	1.0269	1.0175	0.9911	1.4601	− 0.2955	0.1994	0.3356
14	31.6626	1.0230	1.0230	1.0163	0.9867	1.5731	0.2204	0.1972	0.3066
15	38.5851	1.0321	1.0316	0.9951	0.9796	1.4612	− 0.5393	0.1465	0.2265
16	39.5040	1.0298	1.0297	0.9958	0.9807	1.4817	− 0.3004	0.1438	0.2176
17	39.8645	1.0160	1.0172	1.0081	0.9962	1.4683	− 0.0359	0.1444	0.2214
18	42.5676	1.0257	1.0255	1.0011	0.9879	1.4225	− 0.7518	0.1553	0.2521
19	56.3603	1.0261	1.0261	0.9913	0.9814	1.4834	0.108	0.1088	0.164
20	56.6362	1.0290	1.0284	0.9864	0.978	1.4634	0.0865	0.108	0.1563
21	58.6932	1.0198	1.0198	0.9887	0.9914	1.5026	0.5587	0.1241	0.1756
22	61.4805	1.0227	1.0222	0.9919	0.9848	1.4590	0.131	0.1180	0.1744
23	63.6879	1.0380	1.0382	0.9749	0.9671	1.4715	1.4567	0.1126	0.1567
24	86.6989	1.0243	1.0242	0.9852	0.9813	1.4311	− 0.4388	0.0884	0.1293
25	88.3057	1.0241	1.0238	0.9860	0.9818	1.4122	− 0.5719	0.0864	0.1292
26	165.8386	1.0224	1.0188	0.9800	0.9801	1.6134	− 1.008	0.0376	0.0395

In Table 5, 26 arrays of experimental data were presented. It can be seen that all the values of *sl*_*o*1_ and *sl*_*o*2_ were above 1, and all the values of *sl*_*i*2_ were below 1, however, 16 values of *sl*_*i*1_ were above 1 and remaining 10 values were below 1. It can be also seen the situation that both of the values of *sl*_*i*1_ and *sl*_*i*2_ were below 1 was found when the *R*_0_ was comparatively large. Because the turning radius *R*_0_ was so important in analyzing the turning characteristics of the UGV, the data were sequenced by *R*_0_ and ranked from small value to large value.

**Figure 11:**
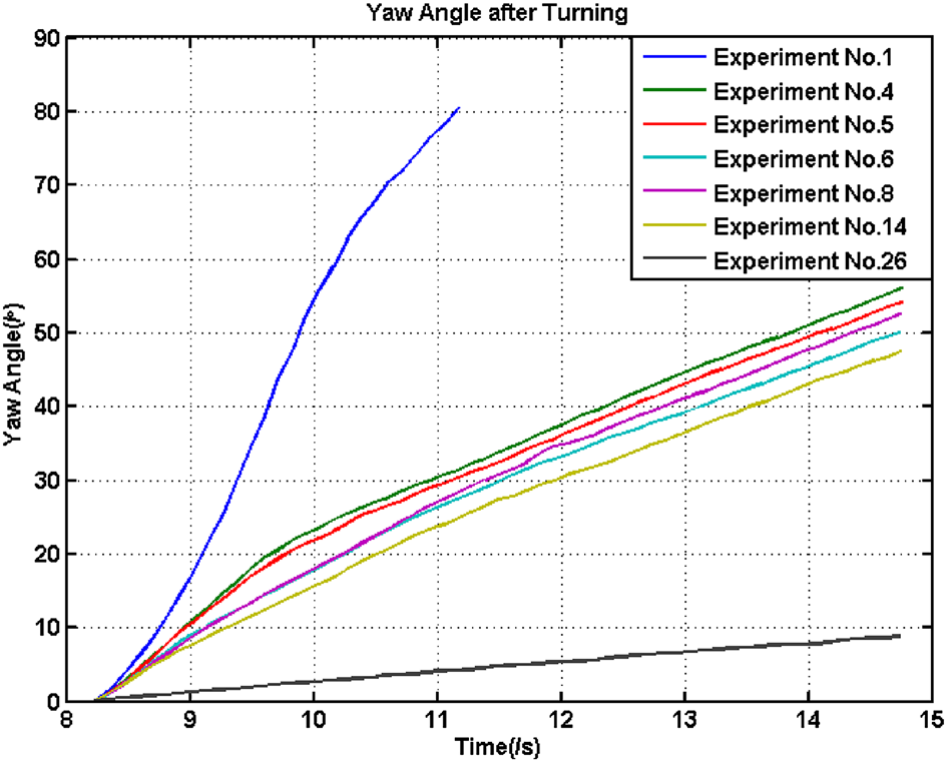
Yaw angle after turning of some experiments.

According to *E*, the majority of these errors were below 1.5%, with the exception of Experiment No.11, and the errors may be positive or negative, which meant that the velocity measurement of the UGV by INS was reliable. In addition, the reliability of the data should also be checked using the calculated width of UGV, *B*_*c*_. The greater the value deviated from the standard value, which was 1.45 m, the more inaccurate the measurement and corresponding calculation. According to the calculation method of this variable, inaccurate calculations may result from many aspects. The first was that the yaw angle was not linearly increasing because of inconsistent slippage occurring for the four wheels, while the second was that the calculation of the difference between the outer and inner trajectories had errors. Figure 11 shows the yaw angle variation of the experiments in which the deviation of *B*_*c*_ was above 0.1 m during the turning stage.

According to Fig. 11, apart from Experiment No. 26, the straightness of the yaw angle curves of other experiments was not high. Especially for the curves of Experiments No. 1, 4 and 5, the curves looked like a parabola instead of a straight line. Although the curve of Experiment No. 26 was straight, the variation in the yaw angle was so small that the final calculation had large errors. Hence, the straightness can determine the accuracy of the calculated turning radius for the majority of situations. Apart from Experiment No.1, all the deviations of *B*_*c*_ were below ± 0.2 m, which meant that a small turning radius can easily induce significant slippage. Because mechanical, assembly and other related errors may induce extra movements, the majority of calculated *B*_*c*_ values were above 1.45 m. Smaller errors of *B*_*c*_, smaller errors and higher accuracy of other calculations. Hence, in the following analysis and calculation, the data shown in Fig. 11 was abandoned in some analyses related to accurate calculations.

**Figure 12:**
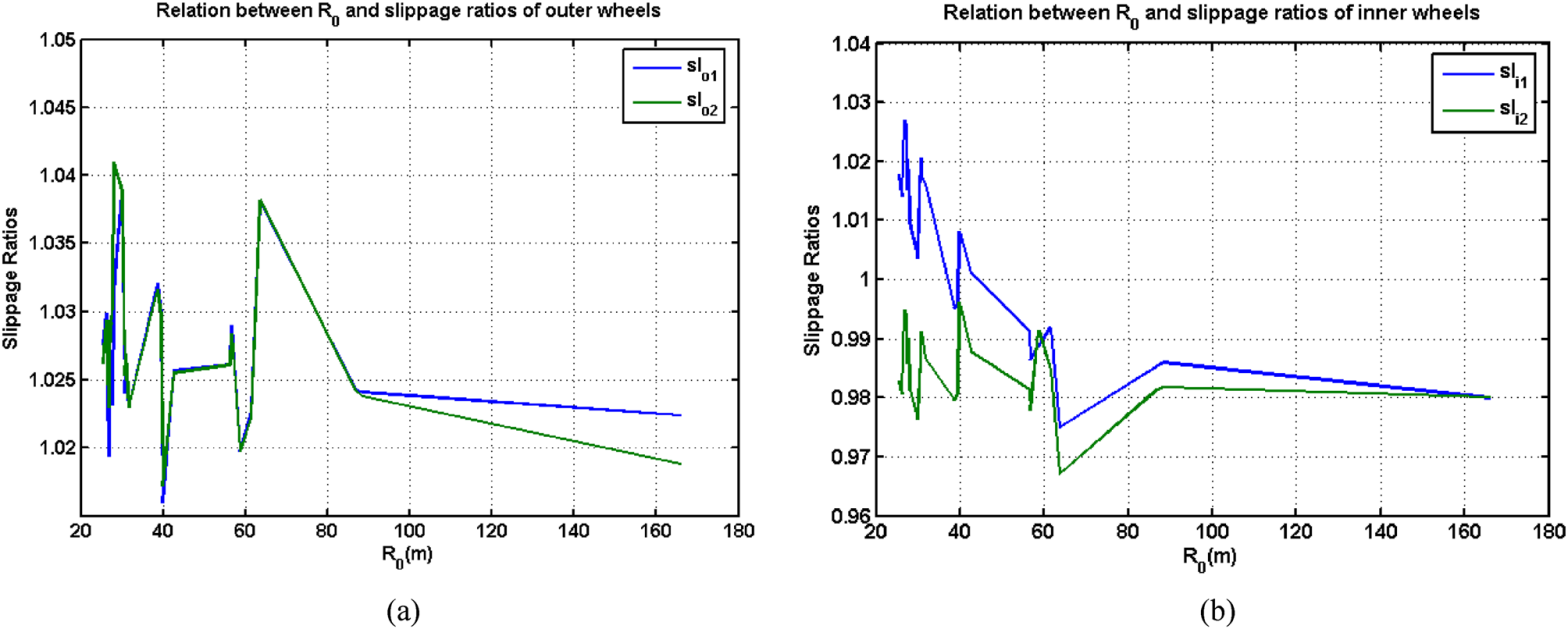
Relations between *R*_0_ and slippage ratios (**a**) outer wheels, (**b**) inner wheels.

For four measurements of slippage ratios, which were *sl*_*o*1_, *sl*_*o*2_, *sl*_*i*1_ and *sl*_*i*2_, it can be noticed that the data had a much greater coincidence. However, when *R*_0_ was below 20 m, these four slippage ratios presented larger differences than other values. Apart from the preceding three values, it can be found that other values had few values. For the remaining 22 arrays of data, the relation between *R*_0_ and the four slippage ratios is shown in Fig. 12.

It can be seen from Fig. 12 that the slippage ratios for the two outer wheels had a high coincidence, and the largest errors were only 0.0036 for the same *R*_0_. The coincidence of slippage ratios for two inner outer wheels was much lower, and the largest errors were achieve 0.035. Corresponding percentage were respectively 0.35% and 3.4%. For the two inner wheels, the inner-rear wheel had a significant velocity decreasing at the starting stage of the turning process, which was coincident with the phenomenon shown in Fig. 7a and Fig. 9a. On the other hand, for these four slippage ratios, during the turning radius *R*_0_ varying from 25.3851 m which was the Experiment 4, the maximum errors for each slippage ratio are 0.0228, 0.0238, 0.0522 and 0.0291. Although the errors were very small, they can greatly affect the calculation of the length of trajectories for each experiment. For the corresponding experiment using this type of large-size UGV, because the turning duration was so short and the distance of the lengths of trajectories between the outer and inner wheels was so limited, only small errors of slippage ratios can induce large calculated errors of the trajectories.

### Model analyses and validation

After checking the reliability and accuracy of the 26 collected arrays of experimental data, these data can be used to perform further analyses combining the model described in “Mathematical model of the large-scale skid-steered four-wheel drive UGV” section. In previous measurements and analyses, a highly accurate INS was employed to confirm the accurate moving velocity. Using the model described in “Mathematical model of the large-scale skid-steered four-wheel drive UGV” section, the four-wheel velocities can be used to derive some more important variables because these velocities were controllable parameters during the operational process. In this part, by means of previous analyses and *u* obtained from INS, slippage characteristics of the UGV were sufficiently considered, and some important relations can also be derived. Then the true turning radius can be calculated only by four-wheel velocities and yaw angle combining the model described in “Mathematical model of the large-scale skid-steered four-wheel drive UGV” section.

**Figure 13:**
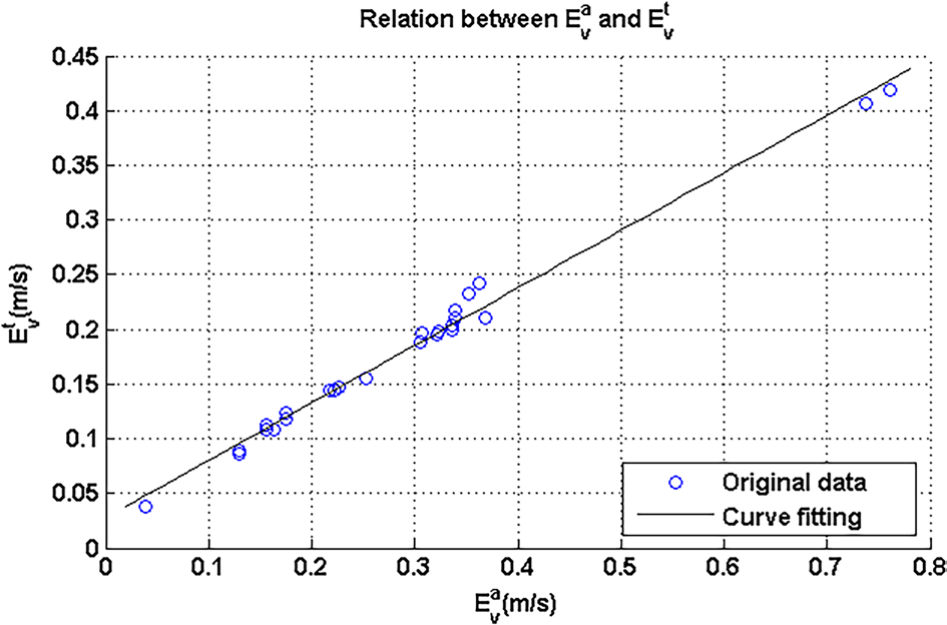
Relation between $E_{v}^{t}$ and $E_{v}^{a}$.

According to Eq. (7) in “Mathematical model of the large-scale skid-steered four-wheel drive UGV” section, each turning radius can be obtained by the widths of UGV, Δ*V*_*x*_ and *u*. In reality, the four-wheel velocities and their differences can be obtained online. The differences of velocities between the outer and inner wheels were $E_{v}^{a}$ in Table 5; however, they should be translated into the standard value, which was $E_{v}^{t}$ in the same table. Hence, it was necessary to seek the relation between these two parameters. Figure 13 showed scatter figure of $E_{v}^{t}$ and $E_{v}^{a}$. To avoid large errors, the preceding three data that had a small turning radius were abandoned.

According to the scatter figure in Fig. 13, it can be observed that the relation was monotone. To obtain a clear and quantitative relation, proper curve fitting was conducted. Obviously, this relation was an approximate linear function. The corresponding curve fitting functions are shown in Eq. (19) and added to Fig. 13.

19 $$E_{v}^{t} = 0.526 \times E_{v}^{a} + 0.02761$$

The corresponding RMSE (Root Mean Square Error) and R-square (Coefficient of Determination) for this curve fitting were respectively 0.9692 and 0.9903, which showed that the curve fitting can sufficiently reflect the relation between these two parameters.

**Figure 14:**
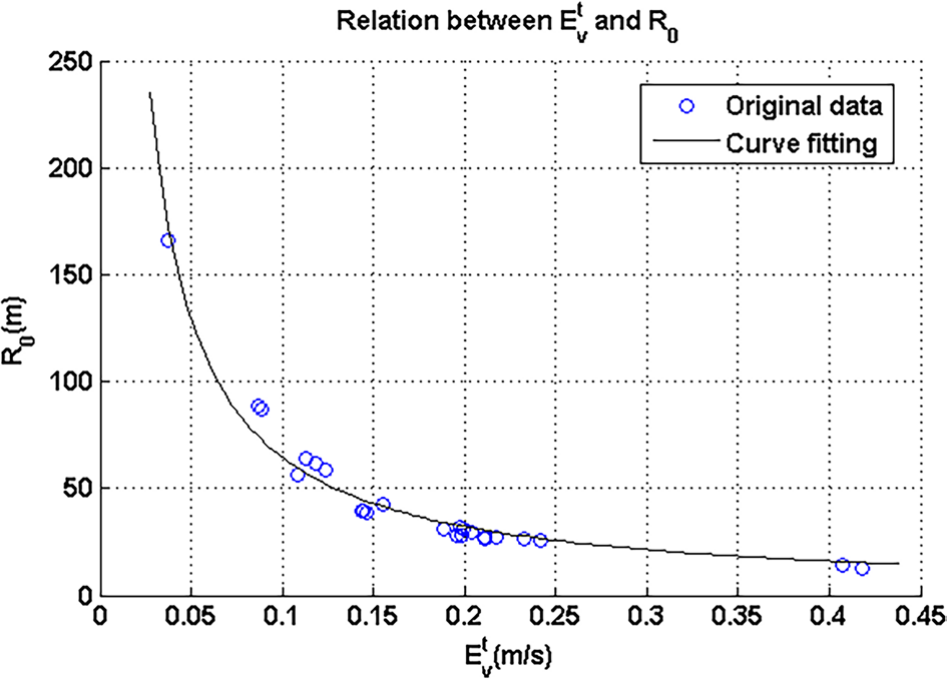
Relation between $E_{v}^{t}$ and *R*_0_.

After velocity difference Δ*V*_*x*_, which was also $E_{v}^{t} ,$ was obtained by collecting wheel velocities, *u* should be considered. This velocity can be considered an average of four-wheel velocities, which should have very small errors in practice. Combining the actual width of the UGV, the corresponding turning radius can be obtained. Additionally, the relation between *R*_0_ and $E_{v}^{t}$, can also be established, and the corresponding scatter is shown in Fig. 14.

It can be noticed from Fig. 14 that the relation was also monotone and was an approximate exponential function. Corresponding curve fitting was also conducted and the curve is shown in Eq. (20) and added in Fig. 14:

20 $$R_{0} { = }6.39 \times E_{v}^{t - 1.004} .$$

The corresponding RMSE and R-square for this curve fitting were respectively 5.723 and 0.9903.

To test the accuracy of the model description and above curve fitting method, Table 6 shows the corresponding calculation results. To avoid large errors, the data shown in Fig. 11 were abandoned.

**Table 6:** Calculation results from model and curve fitting.

No	*R*_0_ (m)	*B*_*c*_ (m)	$E_{v}^{a}$ (m/s)	$E_{v1}^{t}$/Δ*V*_*x*_ (m/s)	*R*_01_ (m)	*E*_*R*01_ (%)	*R*_02_ (m)	*E*_*R*02_ (%)
1	12.9834	1.5341	0.7611	0.4279	13.8553	6.7155	14.9825	15.3972
2	14.0194	1.5311	0.7380	0.4158	14.6749	4.6757	15.4221	10.0053
3	27.2132	1.4611	0.3684	0.2214	27.4079	0.7155	29.0379	6.7052
4	27.7177	1.4796	0.3229	0.1975	29.2906	5.6747	32.5724	17.5149
5	27.9809	1.4523	0.3222	0.1971	29.3283	4.8154	32.6335	16.6278
6	29.8723	1.4841	0.3365	0.2046	31.3889	5.0769	31.4291	5.2116
7	30.6805	1.5378	0.3061	0.1886	32.0212	4.3699	34.1047	11.1608
8	30.7228	1.4601	0.3356	0.2041	31.5270	2.6176	31.5023	2.5372
9	38.5851	1.4612	0.2265	0.1467	40.8179	5.7867	43.8793	13.7207
10	39.5040	1.4817	0.2176	0.1421	41.5037	5.0620	45.3311	14.7505
11	39.8645	1.4683	0.2214	0.1441	41.0975	3.0930	44.6996	12.1289
12	42.5676	1.4225	0.2521	0.1602	43.2433	1.5874	40.1772	− 5.6155
13	56.3603	1.4834	0.1640	0.1138	56.1959	− 0.2917	56.6045	0.4332
14	56.6362	1.4634	0.1563	0.1098	58.2754	2.8943	58.7005	3.6448
15	58.6932	1.5026	0.1756	0.1200	62.5125	6.5072	53.7145	− 8.4826
16	61.4805	1.459	0.1744	0.1193	63.2646	2.9019	53.9997	− 12.1677
17	63.6879	1.4715	0.1567	0.1100	67.0893	5.3407	58.5878	− 8.0080
18	86.6989	1.4311	0.1293	0.0956	83.8528	− 3.2827	67.4562	− 22.1949
19	88.3057	1.4122	0.1292	0.0956	83.6920	− 5.2247	67.4935	− 23.5684

In Table 6, a total of 19 arrays of data and corresponding calculation results were recorded. The preceding three data, *R*_0_, *B*_*c*_ and $E_{v}^{a}$, had the same meanings as in Table 5. In addition, $E_{v1}^{t}$, which was also Δ*V*_*x*_, was the velocity difference calculated based on Eq. (19), *R*_01_ was the turning radius calculated using mathematical model by Δ*V*_*x*_, averaged four-wheel velocities and *B*, and *R*_02_ was the turning radius calculated by direct curve fitting. These two calculations respectively corresponded to Eqs. (7) and (20) in this work. *E*_*R*01_ and *E*_*R*02_ were respectively corresponding errors between *R*_0_, and *R*_01_ and *R*_02_. In this work, these errors were shown in percent form to be clearly and conveniently presented.

According to the results shown in Table 6, using a mathematical model of equations can obtain a more accurate turning radius than using direct curve fitting. The largest error and mean absolute error in the former calculation were respectively 6.7155% and 4.0333%, while those in the latter calculation were respectively 17.5149% and 11.0461%, which meant that the model calculation was reliable. The error distributions had no obvious rule and can be considered random. It can be concluded that the mathematical model described in “Mathematical model of the large-scale skid-steered four-wheel drive UGV” section was reliable and accurate enough, hence, only using the model, four measured wheel velocities and yaw angle, the real time turning radius can be calculated with satisfactory accuracy. However, the direct curve fitting method might had comparatively large errors.

## Conclusion and future works

This work focused on a large-scale skid-steered UGV, which has been widely used in reality. To take knowledge of the characteristics during the turning process, a mathematical model based on geometric characteristics combining the kinematics of this type of UGV was developed. The model analyzed the relations between the turning radius and the velocity difference on both sides, as well as other geometric and mechanical characteristics. Then, an initial experiment that used our self-designed UGV was conducted. Through a series of calculations, it was shown that directly using the velocity difference between the outer and inner wheels cannot obtain reasonable results, because the slippage characteristics made the actual velocity difference between two sides of the UGV smaller than that obtained from directly collected wheel velocities. To obtain reasonable calculation results and further explore the slippage characteristics, an INS with a high accuracy was employed. Then, through a series of tests and comparisons, the slippage levels of the four wheels can be quantitatively measured. Additionally, combining the theoretical derivation of the standard velocities based on the velocity measurement and corresponding calculation using INS, the mathematical model can be validated, and the corresponding comparison showed that the results were better than those obtained directly using curve fitting.

The final results showed that for a given large-scale skid-steered UGV, only using four wheel velocities combined the mathematical model, the real time turning radius can be successfully achieved under the circumstance of sufficiently considering the slippage characteristics. According to a series of experiments, the velocity difference of the wheels between both sides was so small, therefore, accurate measurement of every velocity and the forward moving velocity of the UGV were very important to obtain reasonable analyses and calculation results. In addition, for a large-scale UGV, mechanical, assembly and other related errors may induce more errors, and relatively low maneuverability also prevents accurate measurement results from being obtained, so during the analysis and calculation process, more elements, such as velocity variation and trajectory variation, should be seriously and comprehensively considered. Although this work only considered the low velocity condition, the method can be extended to a high velocity moving condition. Also, the actual turning radius in the experiment was beyond 10 m, which was a normal condition of this large-scale UGV. Too small turning radius may induce more dangerous situation, such as rolling over, or require more power which beyond the common design. Even so, the model, as well as the corresponding analyses and measurement methods, can be used to the normal working condition of this UGV, which will be useful to provide an effective method to analyze and quantitatively evaluate slippage level of this type of UGV. It is anticipated that this work will contribute to this type of UGV related works, regardless of academic research or actual control system design or dead-reckoning work.

## Data Availability

The datasets used and/or analyzed during the current study available from the corresponding author on reasonable request.
